# Known Evolutionary Paths Are Accessible to Engineered ß-Lactamases Having Altered Protein Motions at the Timescale of Catalytic Turnover

**DOI:** 10.3389/fmolb.2020.599298

**Published:** 2020-11-20

**Authors:** Lorea Alejaldre, Claudèle Lemay-St-Denis, Carles Perez Lopez, Ferran Sancho Jodar, Victor Guallar, Joelle N. Pelletier

**Affiliations:** ^1^Biochemistry Department, Université de Montréal, Montréal, QC, Canada; ^2^PROTEO, The Québec Network for Research on Protein, Function, Engineering and Applications, Quebec City, QC, Canada; ^3^CGCC, Center in Green Chemistry and Catalysis, Montréal, QC, Canada; ^4^Barcelona Supercomputing Center, Barcelona, Spain; ^5^ICREA: Institució Catalana de Recerca i Estudis Avancats, Barcelona, Spain; ^6^Chemistry Department, Université de Montréal, Montréal, QC, Canada

**Keywords:** enzyme engineering, epistasis, protein dynamics, protein engineering start-points, TEM-1 beta-lactamase, slow timescales, protein evolution

## Abstract

The evolution of new protein functions is dependent upon inherent biophysical features of proteins. Whereas, it has been shown that changes in protein dynamics can occur in the course of directed molecular evolution trajectories and contribute to new function, it is not known whether varying protein dynamics modify the course of evolution. We investigate this question using three related ß-lactamases displaying dynamics that differ broadly at the slow timescale that corresponds to catalytic turnover yet have similar fast dynamics, thermal stability, catalytic, and substrate recognition profiles. Introduction of substitutions E104K and G238S, that are known to have a synergistic effect on function in the parent ß-lactamase, showed similar increases in catalytic efficiency toward cefotaxime in the related ß-lactamases. Molecular simulations using Protein Energy Landscape Exploration reveal that this results from stabilizing the catalytically-productive conformations, demonstrating the dominance of the synergistic effect of the E014K and G238S substitutions *in vitro* in contexts that vary in terms of sequence and dynamics. Furthermore, three rounds of directed molecular evolution demonstrated that known cefotaximase-enhancing mutations were accessible regardless of the differences in dynamics. Interestingly, specific sequence differences between the related ß-lactamases were shown to have a higher effect in evolutionary outcomes than did differences in dynamics. Overall, these ß-lactamase models show tolerance to protein dynamics at the timescale of catalytic turnover in the evolution of a new function.

## Introduction

Intragenic epistasis describes the non-additive effect of mutations on protein fitness. It has been shown to hinder predictability in protein engineering experiments (Carneiro and Hartl, 2010; Reetz, 2013; Meini et al., 2015; Miton and Tokuriki, 2016; Starr and Thornton, 2016; Yang et al., 2020), highlighting the need for investigation into its causes and effects. In recent years, deep-mutational scanning has been applied to extensively investigate both the impact of single mutations on a given function (Firnberg et al., 2014; Shin and Cho, 2015; Klesmith et al., 2017) and, of particular interest with respect to intragenic epistasis, all possible combinations of double mutants (Olson et al., 2014). However, exhaustive investigation of combinatorial mutations is resource intensive and out of reach for the average-sized protein (a 50 amino acid protein requires a library of 20^50^ = 1.1 × 10^65^ variants). Therefore, focused combinatorial libraries and directed molecular evolution experiments are currently the most useful approach to uncover epistatic interactions (Acevedo-Rocha et al., 2014; Sato et al., 2016; Poelwijk et al., 2019).

Intragenic epistasis comes in different forms. Mutations that individually have a beneficial effect on fitness can act synergistically (positive epistasis) or can be deleterious when combined (negative sign epistasis); on the other hand, deleterious mutations can be beneficial when combined (positive sign epistasis; Starr and Thornton, 2016). The variety of epistatic interactions imposes constraints on mutational pathways leading to a new protein function: pathways that include a node with lower fitness are not evolutionarily accessible whereas specific pathways can be decided by early, positive epistatic effects in evolution (Poelwijk et al., 2007; Salverda et al., 2011; Meini et al., 2015; Miton and Tokuriki, 2016; Yang et al., 2020). Factors known to influence epistasis include direct inter-residue interactions as well as long-range interactions mediated by dynamic networks and protein stability (Miton and Tokuriki, 2016; Starr and Thornton, 2016). Despite increasing evidence that protein dynamics modulate function and fitness (Eisenmesser et al., 2002; Doucet et al., 2007; Osuna et al., 2015; Campbell et al., 2016; Duff et al., 2018; Maria-Solano et al., 2018; Yang et al., 2020), the impact of protein dynamics on intragenic epistasis is not well-understood.

Dynamics are a complex feature of proteins. They encompass motions of varied magnitude and timescales; no single methodology can readily evaluate the full range of motion describing a protein (Maria-Solano et al., 2018; Pandya et al., 2018; Gobeil et al., 2019). Protein dynamics are selected for during natural evolution of enzymes, consistent with dynamics being a determinant of function (Henzler-Wildman and Kern, 2007; Liu and Bahar, 2012; Petrovic et al., 2018). Tracking protein dynamics during the course of experimental evolution has equally revealed correlations between protein dynamics, function and evolution (Johansson and Lindorff-Larsen, 2018; Gardner et al., 2020). Ancestral reconstruction of ß-lactamases revealed that protein motions at fast timescales decreased in conjunction with evolution of specificity (Zou et al., 2015). The directed evolution of a phosphotriesterase to an arylesterase, and back to a phosphotriesterase, revealed that the majority of mutations, although distant from the active site, were epistatic and caused conformational changes (Campbell et al., 2016). Similarly, the additive combination of mutations distant from the active site increased protein motions of inactive proline isomerase CypA, restoring its activity (Otten et al., 2018). Finally, simulations of a beneficial sign epistatic interaction obtained by laboratory evolution of a metallo-ß-lactamase (Tomatis et al., 2008) were experimentally verified to result in increased protein motions on the millisecond to microsecond timescales (Gonzalez et al., 2016).

Evolution is thus shown to modulate protein dynamics; do dynamics modulate the course of evolution? Subjecting to evolution proteins having differing dynamic patterns may reveal alternative evolutionary paths. Alternatively, differing dynamics at the start-point of evolution may not be determinant in evolutionary mechanisms. This might depend on the nature of the protein motions in play: their location and frequency may determine their impact on evolution.

**Figure 1:**
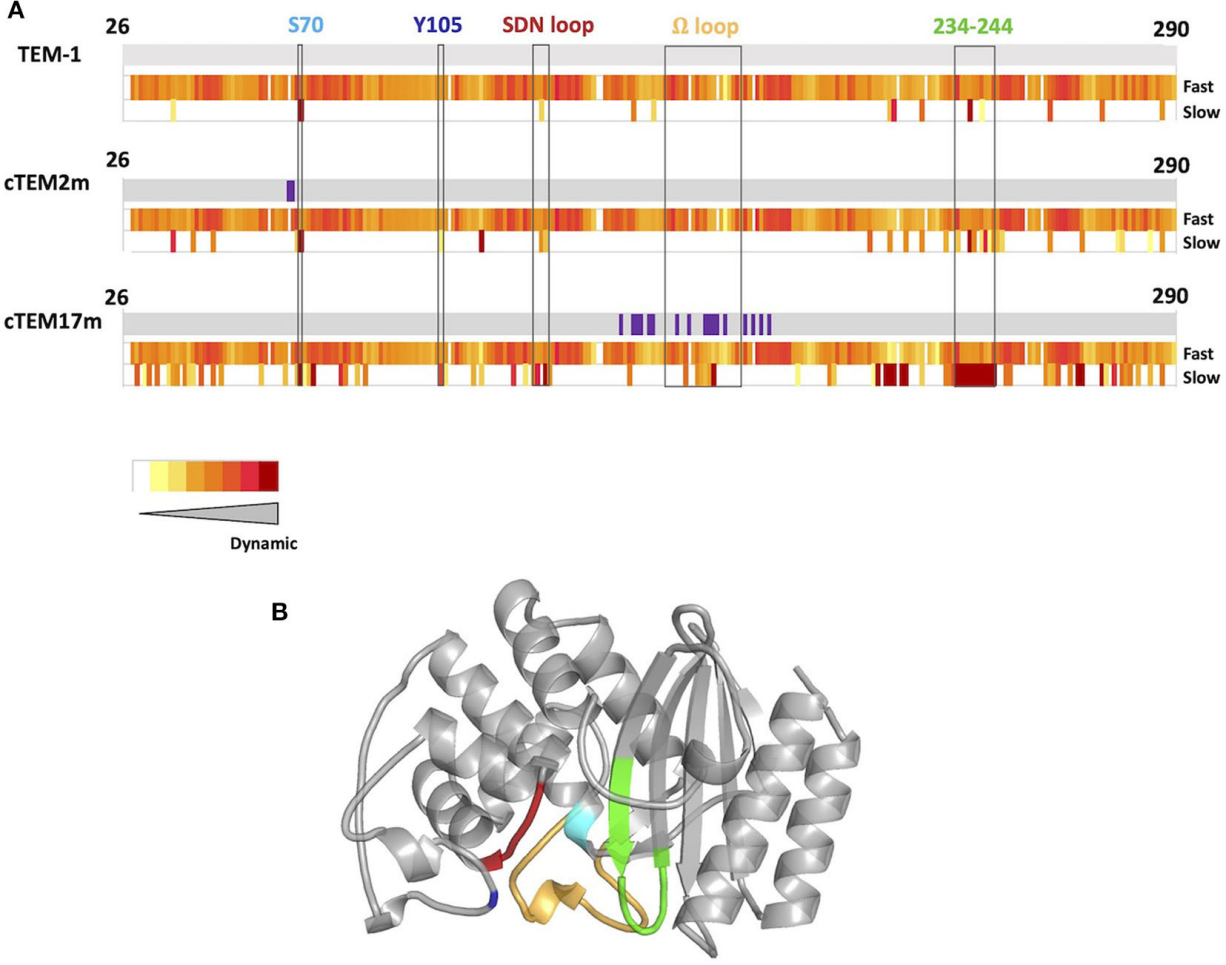
**(A)** Schematic representation of protein dynamics determined at fast time scales (ps-ns) and slow time scales (μs to ms or slower) below sequence diagrams for TEM-1, cTEM-2m, and cTEM-17m (Gobeil et al., 2019). Amino acid substitutions with respect to TEM-1 are shown in purple. The slow timescale coincides with the timescale of catalytic turnover (TEM-1 *k*_cat_ for benzylpenicillin = 450 s^−1^) (Clouthier et al., 2012). **(B)** Structure of TEM-1 highlighting catalytically-relevant regions: S70 (cyan), Y105 (dark blue), SDN loop (red), Ω-loop (yellow), and 234–244 wall (green).

To address this question, we performed the laboratory evolution of two variants of TEM-1 ß-lactamase, a widely-used model for the study of enzyme evolution (Bershtein et al., 2006; Bershtein and Tawfik, 2008; Salverda et al., 2011; Dellus-Gur et al., 2015) that readily evolves toward hydrolysis of new ß-lactam antibiotics (Barlow and Hall, 2002; Salverda et al., 2010). Previous studies have revealed the most prevalent evolutionary paths and shown that the evolution of TEM-1 ß-lactamase is highly constrained due to epistasis (Guthrie et al., 2011; Salverda et al., 2011; Schenk et al., 2013). In addition, high active-site dynamics have been proposed both to promote and to prevent protein evolvability in TEM-1 ß-lactamase (Dellus-Gur et al., 2015; Zou et al., 2015). This suggests that evolving variants having different motions may result in different endpoints, or in different paths to reach the same point. The two specific TEM-1 ß-lactamase variants were chosen because they share similar catalytic activity and substrate recognition, thermal stability, as well as conserved motions at fast (ps-ns) timescales, but they differ greatly in the location and extent of their motions at slow timescales (μs-ms) (Gobeil et al., 2019) (Figure 1).

Mutations E104K/G238S appear in the most efficient evolutionary pathways in natural and directed molecular evolution of TEM-1 toward cefotaxime, exhibiting positive epistasis (Wang et al., 2002; Salverda et al., 2010, 2011; Palzkill, 2018). We therefore introduced mutations E104K and G238S into the two variants of TEM-1 that differ in slow dynamics but exhibit similar catalytic reactivity, to examine the impact of protein dynamics on the evolution of new cefotaximase activity. As for TEM-1, this resulted in synergistic improvement of cefotaximase activity, demonstrating that this evolutionary trajectory remained efficient in different dynamic backdrops. To examine the broader impact of protein dynamics on evolution, we performed three rounds of directed molecular evolution with random mutagenesis to allow exploration of alternative pathways to cefotaximase activity. These revealed that mutations previously reported in the evolution of TEM-1 also appear in the variants with differing slow dynamics. Our results show that TEM-1 ß-lactamase is a robust system where evolvability is compatible with diverse dynamic backgrounds, and where variants possessing slow timescale dynamics that vary in extent and in location can access known evolutionary pathways.

## Materials and Methods

### Site-Directed Mutagenesis

Mutations E104K and G238S were introduced individually and jointly into ß-lactamase constructs TEM-1, cTEM-2m, and cTEM-17m. The ß-lactamase genes were fused to an OmpA signal peptide in 3′ for periplasmic export and cloned into the plasmid pET-24 as previously reported (Morin et al., 2010). Whole plasmid site-directed mutagenesis (Laible and Boonrod, 2009) was done using primers (Sigma Aldrich) designed according to the QuickChange Lightning kit: E104K-F (5′-TATTCTCAGAATGACTTGGTT*AAG*TACTCACCAGTCACAG-3′), E104K-R (5′-CTGTGACTGGTGAGTACT*TAA*CCAAGTCATTCTGAGAATA-3′), G238S-F (5′- GATAAATCTGGAGCC*AGT*GAGCGTGGGTCTC-3′), and G238S-R (5′- GAGACCCACGCTC*ACT*GGCTCCAGATTTATC-3′). Pfu polymerase (Agilent) was used, with reaction conditions according to the manufacturer's recommendations and an extension time of 6 min (~1 min/kb). The reaction product was digested with DpnI (NEB) for 1 h at 37°C to eliminate the template and one-tenth of the reaction was transformed into CaCl_2_-competent *E. coli* XL1-Blue prepared following the Inoue method (Sambrook and Russell, 2006). Where mutagenesis was unsuccessful by that method, mutagenesis was undertaken by overlap extension (Ho et al., 1989). Briefly, the forward primers (above) were employed with the T7- reverse primer (5′- ATGCTAGTTATTGCTCAGC-3′) and the reverse primers (above) with the T7+ forward primer (5′-TAATACGACTCACTATAGGG-3′) in separate PCR reactions with the Phusion polymerase (Thermo Scientific) according to the manufacturer's recommendations. The full-length mutated gene was reassembled from the resulting mutated PCR products in a third PCR reaction using both T7+ and T7- primers. The purified PCR products were subcloned into pET-24 using NdeI and HindIII restriction enzymes (NEB) and DNA ligation kit (Takara). The reaction product was transformed into CaCl_2_-competent *E. coli* XL1-Blue cells. Following selection on Luria-Bertani agar plates containing 50 μg/mL kanamycin, sequences were confirmed by DNA sequencing (IRIC Genomics Platform at Université de Montréal).

### DNA Library Generation

Libraries were generated according to the protocol of Copp et al. (2014). Briefly, random mutagenesis was performed on pET24-cTEM-2m and pET24-cTEM-17m using T7+ and T7- primers with the GeneMorph II kit (Agilent) to obtain a low mutation rate (1-3 mut/kb). Reaction products were sub-cloned into pET-24 as described above, but using T4 DNA ligase (NEB) overnight at 4°C. Following DNA purification using Monarch PCR and DNA purification kit (NEB), half of the reaction was transformed into electrocompetent *E. coli* cells (Lucigen) and spread onto Luria-Bertani agar plates containing 50 μg/mL kanamycin. After overnight incubation at 37°C, the DNA of 10–20 colonies was amplified using 2x PCR Precision Master mix (ABM) and sequenced to verify the mutation rate. DNA sequencing was done with by the Genomic Platform of IRIC or the Genome Quebec Innovation Center at McGill University, Canada. Library size was estimated by counting the resulting colony forming units after overnight incubation at 37°C in Luria-Bertani agar plates containing 50 μg/mL kanamycin. Subsequently, the libraries were pooled, miniprepped using the Monarch Plasmid miniprep kit (NEB) and transformed into *E. coli* BL21(DE3) for expression and screening.

### Directed Molecular Evolution

Libraries were screened on agar plates containing the antibiotic cefotaxime (Sigma-Aldrich) at concentrations 0.08–0.032 μg/mL. Several dilutions of the library were plated on non-selective media to ensure that colony forming units were 10–20-fold higher than the estimated library size. To favor genetic diversity and avoid early evolutionary dead-ends, between 25 and 70 colonies were pooled and selected at each round. Plasmid DNA was extracted with Monarch plasmid miniprep kit (NEB), amplified and used as a template for random mutagenesis to produce the next generation. This process was repeated for a total of three generations. The selection process was tracked by sequencing between 5 and 14 colonies after each selection (IRIC Genomics Platform at Université de Montréal).

### Protein Expression and Purification

Expression and purification of the ß-lactamase variants were performed as in Gobeil et al. (2014). Briefly, 5 mL of an overnight culture were inoculated in 400 mL of ZYP-5052 autoinduction media containing 100 μg/mL kanamycin. After initial growth at 37°C until OD = 0.6, expression was carried out overnight at 22°C. After centrifugation at 3,000 rpm for 30 min, the cell pellet was resuspended in 10 mM Tris-HCl pH 7.0 buffer and lysed using a cell disrupter (Constant Systems). After centrifugation at 20,000 rpm at 4°C for 30 min, the supernatant was filtered through a 0.2 μm filter and injected onto a DEAE-Sepharose Fast Flow column (1.6 × 30 cm). A linear gradient from 10 to 200 mM Tris-HCl pH 7.0 was applied. If purity was <80%, size exclusion chromatography was performed over a Superdex 75 column (1.6 × 55 cm) equilibrated with 50 mM Tris-HCl pH 7.0. Protein concentration and buffer exchange were done in 10 MWCO Ultra Centrifugal Filter Units MilliporeSigma Amicon (Fischer Scientific).

### Minimal Inhibitory Concentration (MIC) Assays

Minimal inhibitory concentration (MIC) assays were performed according to Wiegand et al. (2008) using the agar plate method. Briefly, *E. coli* BL21(DE3) cells expressing the ß-lactamase variants were propagated overnight in Luria-Bertani media containing 50 μg/mL kanamycin. An inoculum of 10^4^ cfu was spotted onto Luria-Bertani agar plates, containing 0.25 mM IPTG and 0.004–1048 μg/mL cefotaxime in 2-fold dilutions. After overnight incubation at 37°C, the plate which had the lowest cefotaxime concentration at which there was no visible growth was considered as the minimal inhibitory concentration. Each construct was assayed in triplicate.

### Enzyme Kinetics

Kinetic parameters for cefotaxime hydrolysis were determined at 27°C in a Cary 100 Bio UV-Visible (Agilent) spectrophotometer, where ε_264nm_ = 7,250 M^−1^cm^−1^ (Clouthier et al., 2012). Cefotaxime concentrations ranged between 12.5 and 200 μM and enzyme concentration 5–70 nM. Kinetic constants were calculated using GraphPad Prism 6 using Michaelis-Menten equation when K_M_ was lower than 150 μM. Otherwise, the Lineweaver-Burk representation was used to calculate kinetic parameters due to inability to saturate the enzyme with CTX, a common issue with cephalosporins (Taibi-Tronche et al., 1996; Vakulenko et al., 1999; Knies et al., 2017; Maryam and Khan, 2017).

### Thermal Stability

Differential scanning fluorimetry was used to determine the melting temperature of the ß-lactamase variants. Protein concentrations of 1−8 μM in sodium phosphate buffer 50 mM pH 7.0 were combined with 2.5 ×, 3.3 ×, and 5 × SYPRO Orange in a final volume of 20 μL. A temperature gradient of 20–95°C with a 0.04°C/s increase was applied in a Light Cycler 480 qPCR instrument (Roche). Fluorescence was measured with λ_exc_ = 483 nm and λ_em_ = 568 nm. Melting temperature values were calculated from the negative derivative of the fluorescence value vs. temperature where the minimum represents the melting temperature (Niesen et al., 2007).

### System Preparation for Computational Analysis

The crystal structures 1XPB for TEM-1, 4MEZ for cTEM-2m, and 4ID4 for cTEM-17m were prepared with Schrodinger's Preparation Wizard. Active-site waters blocking substrate docking were manually removed, taking care to keep the water molecule bridging S70 and E166. Missing side chains were added and sampled through Prime. Hydrogens were added and the protonation states manually checked with the interactive optimizer. For TEM-1, this resulted in H26 and H96 in epsilon protonation, the remaining histidines being delta-protonated. In cTEM-2m, H96 was additionally delta-protonated; it is quite solvent exposed and far from the active site. For cTEM-17m, H26, and H29 were epsilon-protonated and H112 was doubly protonated (thus having a +1 charge). Although the Wizard suggested that K234, immediately next to the nucleophilic S70, should be neutral, we kept it positive as it might be crucial for substrate binding and for stabilizing deprotonation S70 (Atanasov et al., 2000).

We performed Glide docking of cefotaxime using S70 as the grid center and increasing the (grid) inner box diameter to 16 Å to guarantee substrate accommodation. We attempted docking before and after computing quantum charges for the ligands, at the B3LYP DFT and 6/31G^**^ level of theory (Ditchfield et al., 1971; Kim and Jordan, 1994) but obtained analogous results. We thus adopted the classical OPLSAA atom parameters for the ligand since in our experience they might represent better average ones (Shivakumar et al., 2010). Docking used default SP Glide parameters for the three systems resulting in good catalytic positions, with catalytic substrate-serine distances <4 Å. After docking cefotaxime, double mutants were generated with Maestro and the mutated side chains were sampled with Prime prior to performing PELE simulations.

### Protein Energy Landscape Exploration Method (PELE)

Using the cefotaxime-docked structures as input, local PELE explorations were run with the recent AdaptivePELE version (Lecina et al., 2017). PELE is a Monte Carlo protein-ligand sampling procedure capable of accurately mapping ligand migration and induced fit mechanisms (Gilabert et al., 2018). Each simulation involved 20 adaptive epochs of 75 PELE steps each, running on 48 computing cores for a wall clock of ~15 h. The system was described using PELE's default energy function, the 2005 OPLS-AA level of theory with a surface generalized Born model implicit solvent (Yu et al., 2004). For each sampled pose, substrate binding energies were computed using the interaction force field energy: E_complex_ - E_receptor_ - E_substrate_.

### PELE Analysis

In-house Python scripts were used to identify poses with the lowest interaction energies where the catalytic S70 was at a distance of <3.5 Å from C3 of the cefotaxime ß-lactam ring. Scripts are available upon request. Visual inspection of the poses as well as identification of productive poses was done using VMD (Humphrey et al., 1996). The salt-bridges plugin in VMD was used to identify interactions. This considers that a salt-bridge is formed when any oxygen atom of a residue identified as acidic and any nitrogen atom of a residue identified as basic are at a cutoff distance of <3.2 Å in at least one frame. Distance between residue 104 and P167 was calculated using the Tk console of VMD with a freely available script at https://github.com/ipudu/useful-vmd-scripts/blob/master/distance.tcl. Distances were calculated between the center of mass of two residues, as well as between the α-carbons. Active site volume was calculated using F-Pocket (Le Guilloux et al., 2009).

## Results

### Positive Epistasis Is Maintained in a Known Evolutionary Path Despite Altered Protein Dynamics

**Figure 2:**
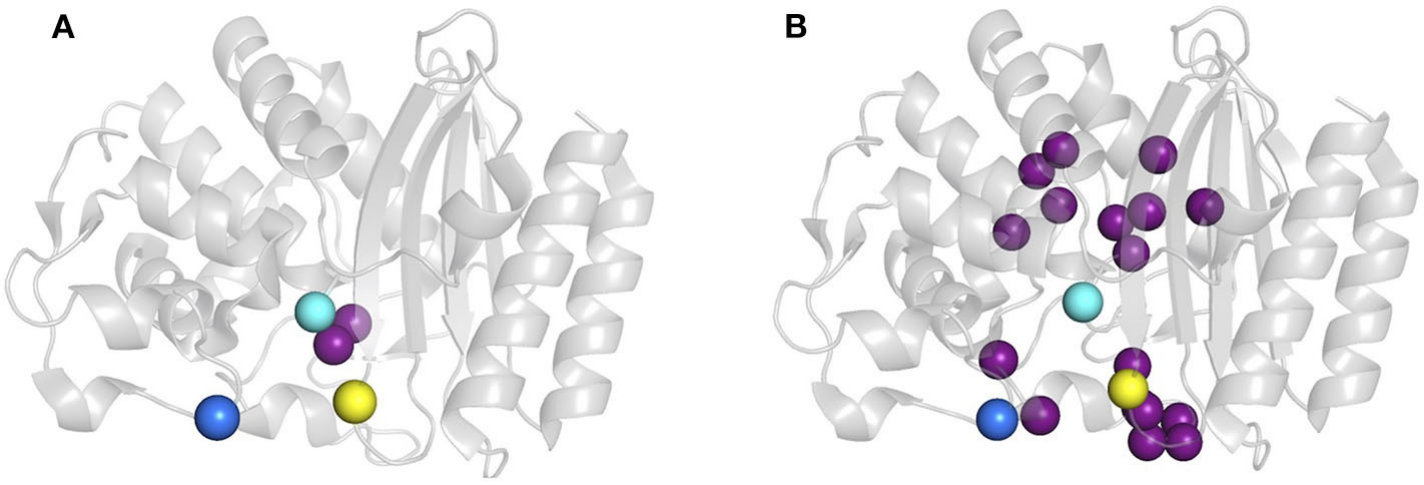
Location of mutations E104K and G238S in host ß-lactamases. **(A)** cTEM-2m (PDB 4mez) and **(B)** cTEM-17m (PDB 4id4). Mutated residues with respect to TEM-1 are highlighted as purple spheres and the catalytic serine 70 is in cyan, E104K in dark blue and G238S in yellow.

Protein dynamics on slow timescales (μs to ms or slower) see large conformational rearrangements associated with ligand-binding and substrate turnover. We previously reported two variants of TEM-1 ß-lactamase where functionality is maintained despite sequence differences marked differences the magnitude and location of slow protein motions (Figure 1) (Gobeil et al., 2014, 2019). Variants cTEM-2m and cTEM-17m include, respectively, two and 17 substitutions in the active site region. The substitutions of cTEM-2m are M68L and M69T, the immediate neighbors of the nucleophilic S70; those of cTEM-17m are on an opposite face of the active site, within and flanking the catalytic Ω-loop (Figure 2). In contrast to TEM-1, where only 13 residues display slow motions near the frequency of turnover [μs to ms or slower, where the *k*_cat_ of TEM-1 for benzylpenicillin is 450 s^−1^ (Clouthier et al., 2012)], variant cTEM-2m exhibits slow motions in 29 residues; these are predominantly located in the active-site cavity at the catalytically relevant Y105 and SDN loop but exclude the Ω-loop. Variant cTEM-17m differs yet more strikingly, with slow motions mapped to 82 residues that broadly span the enzyme and include the Ω-loop (Figure 1) (Gobeil et al., 2019). Nonetheless, their catalytic efficiencies (*k*_cat_/K_M_) are within one order of magnitude of the native TEM-1 [(Clouthier et al., 2012; Gobeil et al., 2019) and Table 1]. Conservation of their kinetic properties correlates with conservation of their fast motions (ps-ns), despite wide-ranging alterations of their motions on the timescale of catalytic turnover (Henzler-Wildman and Kern, 2007).

**Table 1:** Cefotaximase activity *in vitro* and in *E. coli*, and thermostability of host ß-lactamases TEM-1, cTEM-2m, cTEM-17m, and their corresponding E104K/G238S variants.

	**K_**M**_**	***k*_**cat**_**	***k*_**cat**_/K_**M**_**	**MIC**	**T_**m**_**
	**(μM)**	**(s^**−1**^)**	**(mM^**−1**^ s^**−1**^)**	**(μg/mL)**	**(°C)**
TEM-1	1,500 ± 900	1.0 ± 0.5	0.68 ± 0.53	0.03	49.9 ± 0.3
TEM-1 E104K	750 ± 560 (0.5×)^a^	1.0 ± 0.6 (1×)	1.3 ± 1.2 (2×)	0.1	49.4 ± 0.1
TEM-1 G238S	240 ± 80 (0.2×)	30 ± 8 (30×)	130 ± 100 (190×)	0.6	43.8 ± 0.1
TEM-1 E104K/G238S	150 ± 50 (0.1×)	33 ± 9 (33×)	220 ± 90 (320×)	130	43.5 ± 0.1
cTEM-2m^b^	240 ± 40	0.02 ± 0.01	0.083 ± 0.044	0.02	49.6 ± 0.2
cTEM-2m E104K	680 ± 170 (3×)	0.3 ± 0.1 (15×)	0.44 ± 0.25 (5×)	0.02	47.5 ± 0.4
cTEM-2m G238S	290 ± 40 (1.2×)	1.4 ± 0.3 (70×)	4.8 ± 1.4 (60×)	0.04	42.3 ± 0.2
cTEM-2m E104K/G238S	77 ± 10 (0.3×)	3.8 ± 0.2 (190×)	49 ± 7 (600×)	0.09	42.0 ± 0.4
cTEM-17m^c^	260 ± 100	0.14 ± 0.04	0.54 ± 0.26	0.03	49.0 ± 0.8
cTEM-17m E104K	920 ± 90 (4×)	3.1 ± 0.5 (20×)	3.4 ± 0.7(6×)	0.02	48.3 ± 0.3
cTEM-17m G238S	730 ± 290 (3×)	23 ± 8 (160×)	32 ± 17 (60×)	43	48.0 ± 0.5
cTEM-17m E104K/G238S	130 ± 30 (0.5×)	25 ± 3 (180×)	190 ± 50 (350×)	109	46.9 ± 0.5

a *Fold increase relative to the respective host ß-lactamase is given in parentheses. Changes equal to or >1 order of magnitude are highlighted in red*.

b *Values taken from Gobeil et al. (2014)*.

c *Values taken from Clouthier et al. (2012)*.

The synergistic mutations E104K/G238S are prevalent in cefotaxime-resistant clinical isolates and consistently appear in directed molecular evolution experiments (Barlow and Hall, 2002; Salverda et al., 2010; NCBI, 2018). These mutations were introduced into the two TEM-1 variants to determine whether the E104K/G238S mutational pathway to cefotaxime resistance is accessible in the context of altered active-site residues and slow motions. The impact of the two and 17 mutations of variants cTEM-2m and cTEM-17m on the newly inserted E104K/G238S mutations was evaluated by determining the *in vitro* kinetics of cefotaxime hydrolysis (Table 1). In all cases, inclusion of E104K/G238S procured remarkable increases in catalytic efficiency (~600-fold in cTEM-2m; ~360-fold in cTEM-17m), comparable to the ~320-fold increase observed for TEM-1 E104K/G238S. These results immediately demonstrate that, despite exhibiting unique and widespread patterns of increased slow dynamics throughout the active-site (Figure 1), cTEM-2m and cTEM-17m can evolve cefotaximase activity via the same epistatic interaction as TEM-1 ß-lactamase (Table 1).

Variant cTEM-17m is the most genotypically distant from TEM-1 (17 substitutions) and differs most in the range and extent of its slow dynamics, yet inclusion of mutations E104K/G238S had essentially the same effect on catalytic efficiency (190 mM^−1^ s^−1^, relative to 220 mM^−1^ s^−1^ in TEM-1, Table 1); variant cTEM-2m was only 4-fold less efficient. Catalytic turnover (*k*_cat_) was strongly improved by inclusion of mutations E104K/G238S in all three settings: a ~180-fold increase in cTEM-2m and cTEM-17m, significantly greater than the ~30-fold increase in TEM-1. This was accompanied by a modest increase in productive binding to cefotaxime: K_M_ was reduced by ~2-3-fold in cTEM-2m and cTEM-17m, which both had higher affinity at the outset, compared to ~10-fold in TEM-1. Although mutations E104K/G238S have been found to be destabilizing in TEM-1 and are often accompanied with the stabilizing mutation M182T (Wang et al., 2002), it has been reported that thermostability is not a major driving force in the evolution of CTX resistance in TEM-1 ß-lactamase (Knies et al., 2017). It is interesting to note that inclusion of mutations E104K/G238S decreased thermostability by ~7°C in both TEM-1 and cTEM-2m; despite cTEM-17m displaying the most widespread slow dynamics, its melting temperature was hardly affected by inclusion of mutations E104K/G238S (~2°C decrease).

Insertion of the individual E104K and G238S substitutions revealed clear positive epistasis on cefotaximase specific activity in the three host ß-lactamases (Table 1). Inclusion of G238S procured 10- to 100-fold greater improvement to specific activity against cefotaxime than E104K in each host, consistent with the prevalence of the G238S substitution in clinical isolates/evolution toward cephalosporins (Barlow and Hall, 2002; Salverda et al., 2010; NCBI, 2018). Most of the catalytic improvement resulted from increased *k*_cat_ due to G238S (30 to 160-fold). In contrast, the effects of the individual mutations on K_M_ were modest, whether in improving K_M_ in TEM-1 (0.2 to 0.5-fold) or weakening it in cTEM-2m and cTEM-17m (1.2 to 4-fold) (Table 1). Indeed, sign epistasis was observed for K_M_ in cTEM-2m and cTEM-17m; in both cases, one or both substitutions weakened K_M_ and their combination had a favorable effect on K_M_. This demonstrates that marked synergy exists between substitutions E104K and G238S in all three host ß-lactamases yet the pattern of the synergy is modulated by the differences between the hosts.

At a glance, the MIC values against CTX of the individually and doubly substituted variants did not show a clear correlation with the kinetic parameters (Table 1). A closer look shows similar trends in improvement of specific activity and increased MIC for the TEM-1 series and the cTEM-17m series. In both cases, G238S had the greatest individual effect and combination of E104K and G238S showed clear positive epistasis, similar to effects on specific activity. This relation did not hold in cTEM-2m. cTEM-2m has the lowest *k*_cat_ of the three host ß-lactamases; although the inclusion of E104K/G238S increased its *k*_cat_ 200-fold, reflected in its 600-fold improvement in catalytic efficiency, MIC increased only ~4-fold (Table 1). We hypothesize that catalytic turnover is a limiting factor: in all cases, only when *k*_cat_ is > 20 s^−1^ are important increases in MIC observable. Expression of all variants in *E. coli* was similar, but factors inherent to the *in vivo* MIC assay such as altered mRNA or protein stability, folding or periplasmic export may play an additional role. For instance, the correlation between MIC and activity assays increased when kinetic stability, that is aggregation and degradation in native conditions, was considered for BcII metallo-ß-lactamase (Meini et al., 2015). In the current study, thermostability is unlikely to be in play since TEM-1 and cTEM-2m showed a similar loss of thermostability upon inclusion of E104K/G238S (Table 1).

Overall, *in vitro* assays demonstrate that the E104K/G238S combination acts synergistically in all three host ß-lactamases. This is observable *in vivo* through MIC assays only for host ß-lactamases TEM-1 and cTEM-17m, indicating that the improvement in catalytic function in host cTEM-2m was insufficient to overcome microbial inhibition with CTX. These considerations will influence the outcome of directed molecular evolution, where cefotaximase function is selected via an *in vivo* process.

### Rationalization of Catalytic Improvement Through Flexible Molecular Docking

The inclusion of E104K and G238S resulted in a similar extent of catalytic improvement in the three host ß-lactamases despite the variation in active-site sequence and dynamics at the timescale of cefotaxime turnover. The mechanism by which these mutations provide a synergistic effect in TEM-1 is not yet fully understood (Salverda et al., 2010; Miton and Tokuriki, 2016). Here, we examined whether the mechanism of catalytic improvement was conserved by performing Protein Energy Landscape Exploration (PELE) (Borrelli et al., 2005) simulations. PELE is a Monte Carlo approach that combines random localized perturbations of the substrate and protein backbone with side-chain sampling and minimization cycles. Such a combination has been shown to provide robust descriptions of substrate migration and active-site induced-fit (Acebes et al., 2016; Carro et al., 2019). This is key, as previous research has found that docking to a single structure does not accurately predict increases in catalytic efficiency (Hart et al., 2016). Here, the rate-limiting step for cephalosporin hydrolysis is the acylation process (Saves et al., 1995), which is the first step in the reaction mechanism. As a result, correlation of the enzyme-substrate catalytic distances with the Michaelis-Menten kinetic parameters is pertinent.

**Figure 3:**
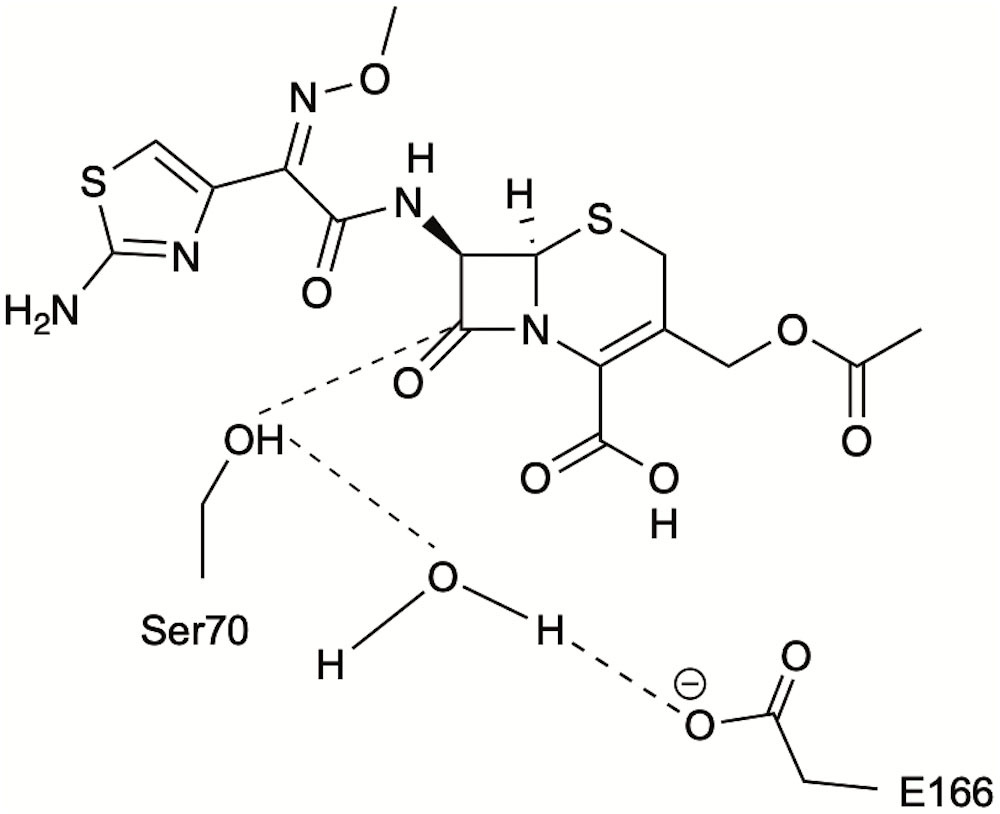
Schematic representation of the catalytic distances monitored during PELE simulations for identification of catalytically relevant frames: S70 hydroxyl oxygen to CTX(C3), S70 hydroxyl hydrogen to catalytic water, and E166 carboxylate oxygen to catalytic water.

**Figure 4:**
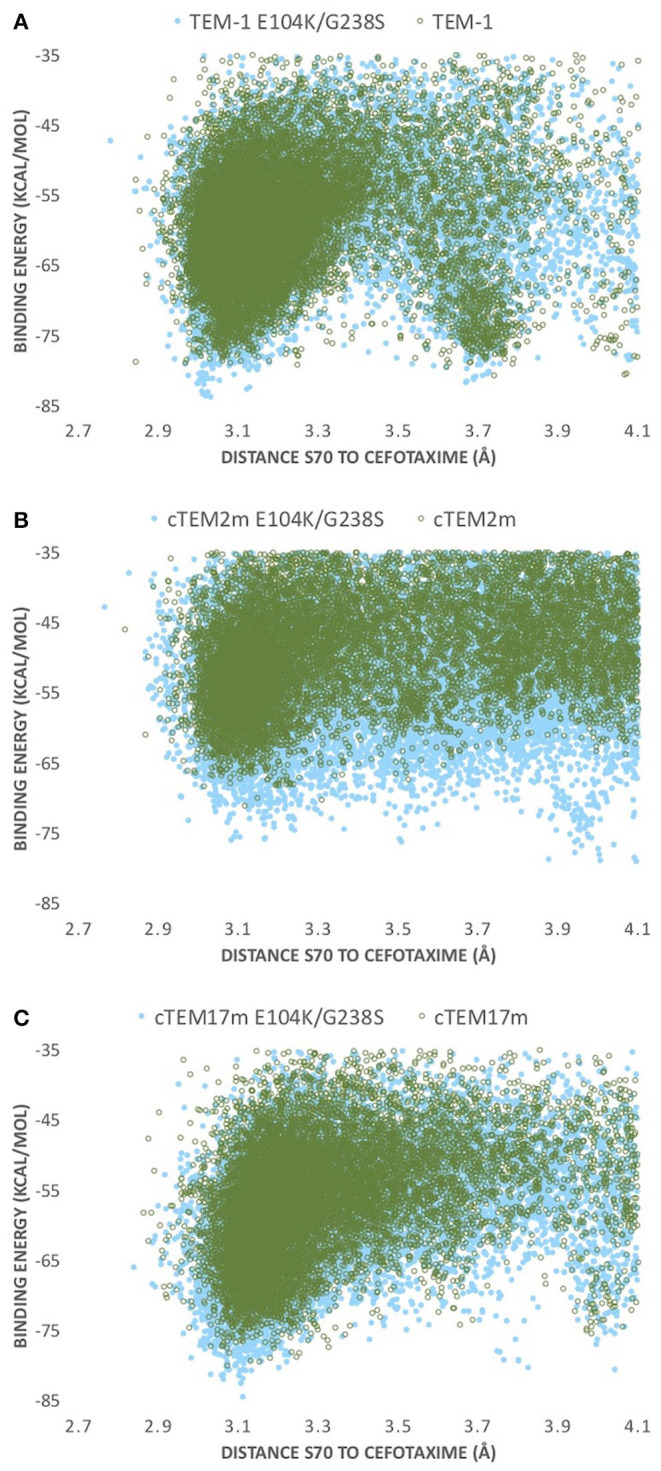
PELE energy profiles for the host ß-lactamases **(A)** TEM-1, **(B)** cTEM-2m, and **(C)** cTEM-17m (empty green circles) and their respective E104K/G238S variant (full blue circles). Binding energy is plotted against distance of S70 hydroxyl oxygen to CTX(C3).

Our simulations identify such a correlation with catalytic efficiency. Each of the E104K/G238S variants presents significantly more favorable (lower) interaction energies at the catalytic distance monitored (distance from catalytic S70 to C3 carbon in cefotaxime; Figure 3) than their respective non-mutated hosts, reflecting the improved catalytic efficiency of each E104K/G238S variant (Figure 4). In TEM-1, cTEM-17m and their E104K/G238S variants, the energy minima observed at catalytically relevant distances (~3 Å) are sharper, showing a more productive exploration of the active site by the substrate than in cTEM-2m and its double mutant (Figure 4). The non-mutated TEM-1 has a second energy minimum at ~3.7 Å (almost degenerate with that at ~3 Å). Inclusion of E104K/G238S in TEM-1 produced a greater stabilization of the catalytically productive minimum than the non-productive minimum, contributing to the increase in catalytic efficiency. This is consistent with previous results, where mutations E104K/G238S were shown to reduce the incidence of non-productive conformations (Hart et al., 2016).

The conformers corresponding to the lowest interaction energies and shortest distances between the catalytic S70 and the CTX ß-lactam ring for each variant were defined as catalytically productive (Figure 3). This excluded binding in catalytically disfavored conformations, such as conformers displaying favorable binding energy but Ser-CTX distances incompatible with catalysis (>3.7 Å) (Figure 4). They were extracted using the lowest interaction energies where the catalytic S70 was within 3.5 Å of C3 in the CTX ß-lactam ring. The average catalytic distances are similar (Table 2). Then, the 50 best frames with <3.5 Å between the catalytic water and the hydroxyl oxygen of S70 as well as a carboxylate oxygen in E166 were selected, again excluding any catalytically disfavored conformation (Figure 3). The predicted binding energies of the catalytically productive structures improved upon inclusion of E104K/G238S into each of the three host ß-lactamases (Table 2). Despite a 2-fold greater improvement in host cTEM-2m (average 6.1 kcal/mol) than in hosts TEM-1 and cTEM-17m (average ~3 kcal-mol), the predicted binding energy of cTEM-2m E104K/G238S was weaker than the other double mutants. This correlates with its lower catalytic efficiency and poor performance in MIC assays (Table 1). Further analysis revealed a new salt-bridge in each of the double mutants (Supplementary Table 1). The E104K-E240 salt bridge appeared in seven out 50 poses in TEM-1 E104K/G238S, 40 out of 50 poses in cTEM-2m E104K/G238S and 12 out of 50 poses in cTEM-17m E104K/G238S. Although it did not appear in all poses, it signals a potential contribution to stabilizing catalytically relevant conformations.

**Table 2:** Analysis of the 50 catalytically productive variants having the lowest interaction energy.

		**Binding Energy (kcal/mol)**	**Distances** **(Å)**				**E104K-E240 salt-bridge (%)**
			***S70-CTX(C3)***	***S70-catalytic water***	***E166-catalytic water***	***104-P167***	
TEM-1	Average	−76.3	3.1	2.7	3.1	6.7	N/A
	*Max*	−74.9	3.5	2.8	3.3	7.3	
	*Min*	−78.8	2.8	2.6	2.9	5.7	
TEM-1 E104K/G238S	Average	−79.1	3.1	2.7	3.2	5.6	14
	*Max*	−77.0	3.5	2.8	3.5	6.8	
	*Min*	−83.7	2.9	2.7	3.0	5.0	
cTEM-2m	Average	−65.7	3.2	2.7	3.2	7.6	N/A
	*Max*	−64.1	3.5	2.7	3.5	8.1	
	*Min*	−70.3	3.0	2.6	3.0	7.1	
cTEM-2m E104K/G238S	Average	−71.8	3.2	2.7	3.1	5.8	80
	*Max*	−69.9	3.4	3.4	3.4	6.8	
	*Min*	−76.0	3.0	2.6	2.4	5.1	
cTEM-17m	Average	−76.4	3.2	2.7	2.6	6.8	N/A
	*Max*	−74.9	3.4	2.7	3.2	7.1	
	*Min*	−80.1	3.0	2.6	2.4	6.4	
cTEM-17m E104K/G238S	Average	−79.4	3.1	2.7	2.6	5.5	24
	*Max*	−77.9	3.2	2.9	3.2	6.1	
	*Min*	−84.5	3.0	2.7	2.4	5.2	

*N/A, not applicable*.

In addition, the inclusion of E104K/G238S decreased the distance between residue 104 and P167 in all variants, by an average of 1.4 Å (1.1–1.8 Å; Table 2). P167 belongs to the Ω-loop and is the neighbor of the catalytic E166; its van der Waals interaction with K104 has been proposed to stabilize catalytically relevant conformations (Hart et al., 2016). We note that this stabilization did not significantly modify the backbone distances between residue 104 and P167.

It is interesting to note that cTEM-17m has a larger active site pocket (5,000 Å^3^) than TEM-1 (2,200 Å^3^) and cTEM-2m (2,600 Å^3^) (Table 2). The active-site cavity size was greatly reduced in cTEM-17m E104K/G238S (2,320 Å^3^), bringing it in the range of TEM-1 E104K/G238S (2,350 Å^3^) and cTEM-2m E104K/G238S (1,980 Å^3^) which showed smaller variation in cavity size with their respective hosts. The observations made with cTEM-17m demonstrate that variation in the active site cavity volume is tolerated in TEM-1 variants.

### Directed Molecular Evolution Toward Cefotaxime Hydrolysis in cTEM-2m and cTEM-17m

Overall, the combined E104K/G238S substitutions consistently led to important improvements in catalytic efficiency in the three hosts, as reflected in the more favorable energies predicted for their cefotaxime complexes. Nonetheless, the effects of the E104K/G238S substitutions on MIC in *E. coli* were variable. Importantly, the individual E104K or G238S substitutions had significantly different effects on MIC in the three hosts, potentially because low catalytic turnover (*k*_cat_) is a limiting factor upon selection for cefotaxime resistance in bacteria. This led us to question whether the evolutionary paths to cefotaxime resistance available to these three hosts could differ, should epistatic interactions differ significantly due to initial slow motions.

Having demonstrated that the prevalent E104K/G238S evolutionary solution to cefotaxime resistance in TEM-1 ß-lactamase is valid in variants cTEM-2m and cTEM-17m, we investigated whether other known evolutionary paths toward cefotaxime resistance in TEM-1 (Barlow and Hall, 2002; Weinreich, 2006; Salverda et al., 2010; Palzkill, 2018) are also accessible to cTEM-2m and cTEM-17m host ß-lactamases by performing three rounds of random mutagenesis and selection. TEM-1 has served in the past as a robust model to describe new mutagenesis methodologies (Fujii et al., 2004; Firnberg and Ostermeier, 2012), deep-mutational scanning (Firnberg et al., 2014; Stiffler et al., 2015) and to test protein evolution hypotheses (Bershtein and Tawfik, 2008), where screening vast libraries for improved cefotaximase activity has proven practical and informative. As a result, a large body of information describing evolutionary paths to higher cefotaximase activity in TEM-1 is available. Prevalent mutations known to confer cefotaxime resistance occur in the Ω-loop region (R164S, R164H, I173V), and its neighboring regions: 234–244 region (A237T, G238S, E240K) and E104K. Some of these are known to act synergistically as E104K and G238S do, E240K is often encountered in combination with R164S or G238S and A237T with R164S.

We thus investigated evolutionary paths to cefotaximase activity using cTEM-2m and cTEM-17m ß-lactamases as starting points, to determine whether they include the same substitutions as TEM-1 ß-lactamase or if distinct ones are selected. Libraries consisting of >10^5^ variants with a mutation rate of 1–5 mutations/1,000 bp/generation were screened over three generations (Table 3). Each generation saw the libraries plated on several concentrations of cefotaxime. Colonies were collected and pooled at the lowest cefotaxime concentration where significant selection was observed. This strategy allowed for inclusion of moderate sequence diversity in each subsequent round of mutagenesis, thereby minimizing the occurrence of evolutionary dead ends. The mutational process was tracked by sequencing randomly picked colonies throughout the process of laboratory evolution.

**Table 3:** Mutation rate and library size for three rounds of directed molecular evolution, prior to selection.

	**Mutation rate/1,000 bp**		**Library size (CFU)^a^**	
	**cTEM-2m**	**cTEM-17m**	**cTEM-2m**	**cTEM-17m**
Generation 1	2.6 (10)^b^	3.3 (16)	1.5 × 10^5^	6.0 × 10^5^
Generation 2	4.9 (13)	4.0 (10)	3.0 × 10^5^	1.7 × 10^5^
Generation 3	9.4 (12)	7.5 (10)	3.7 × 10^4^	2.6 × 10^5^

a *CFU: colony-forming units*.

b *The number of variants sequenced is in parentheses*.

Fixation of one variant was identified following two rounds of evolution for cTEM-2m: variant cTEM-2m (**L40F**/**G116S**/T160T/**P167L**/**R178C**; where non-synonymous substitutions are in bold) was observed in 7 out of 9 colonies sequenced and was the sole variant identified after selection of the third generation (Table 4). PCR biases during the mutagenic process were ruled out as that variant was not predominant prior to selection (one out of 13 sequences, Supplementry Table 2).

**Table 4:** Mutations identified upon screening cTEM-2m libraries against 0.016 μg/mL CTX.

**cTEM-2m**	**40**	**57**	**91**	**104**	**116**	**118**	**160**	**167**	**169**	**170**	**178**
Generation 1				**E>V**		**T>A**					
										**N>I**	
		**I>P**									
Generation 2			**L>V**		**G>S**		T>T	**P>L**	L>L		**R>C**
	**L>F**				**G>S**		T>T	**P>L**			**R>C**
Generation 3	**L>F**				**G>S**		T>T	**P>L**			**R>C**

*Non-synonymous mutations are highlighted in bold*.

Three generations of evolution were required to observe fixation of a variant of cTEM-17m (Table 5). Variant cTEM-17m (P62P/A135A/**K158N**/R164R/**K192N**/K234K) was identified in 10 out of 14 colonies sequenced. Of special interest is that mutations E104K and G238S were individually observed following the selection of the second generation, accompanied by one or more non-synonymous mutations, but neither became dominant following selection of the third generation (Table 5).

**Table 5:** Mutations identified upon screening cTEM-17m libraries against 0.016 μg/mL CTX.

**Generation**	**42**	**53**	**62**	**70**	**78**	**91**	**92**	**100**	**104**	**125**	**134**	**145**	**158**	**164**	**170**	**182**	**185**	**188**	**190**	**192**	**197**	**228**	**234**	**238**	**239**	**246**	**249**	**253**	**254**	**256**	**275**
1																			L>L								**A>C**				
															N>N															**K>M**	
						**L>F**										T>T															
					G>G						A>A					T>T		**S>G**													
2			P>P							A>A																			**K>R**		
				S>S								P>P																			
				S>S													L>I										L>L				**R>G**
			P>P							A>A														**G>S**	**E>G**						
			P>P							A>A														**G>S**		**I>V**					
			P>P						**E>K**																**E>G**			**G>S**			
3			P>P							A>A			**K>N**	R>R						**K>N**			K>K								
							**G>D**	N>N																							
			P>P																	**K>N**											
	A>A												**K>N**								**E>K**										
		**S>N**								A>A				R>R								G>G							**K>R**		

*Bold value indicates non-synonymous mutations*.

### Fixated Variants in Directed Evolution Belong to Known Evolutionary Paths

Sampling evolutionary paths toward cefotaxime resistance showed that cTEM-17m could access known prevalent mutations in directed evolution and clinical isolates. The experiment was continued until a variant was fixated in cTEM-2m and cTEM-17m evolution. The fixated variants were sub-cloned and transformed to confirm cefotaxime resistance. Predominance of a variant indicates that these clones contain mutations that provide an advantage with respect to the other clones in the pool when the mutational load is increased. The location and prior reports on these mutations is discussed in this section.

The predominant cTEM-2m (**L40F**/**G116S**/T160T/**P167L**/**R178C**) variant includes two non-synonymous mutations in the Ω-loop that had previously been reported in clinical isolates or directed molecular evolution experiments in TEM-1 (Fujii et al., 2004; Schenk et al., 2013; NCBI, 2018). In particular, P167 is the neighbor to catalytic residue E166 and is a key residue for folding in TEM-1 (Vanhove et al., 1996). The P167L substitution observed here was previously found upon selection for CTX and ceftazidime hydrolysis, accompanied by further mutations known to confer ceftazidime resistance (Schenk et al., 2013). Mutation of R178 to A has been observed in clinical isolate TEM-178, and to C in a multiply-mutated variant evolved toward ceftazidime resistance(Fujii et al., 2004). It is interesting to note that both previously reported P167L and R178C variants containing these mutations were accompanied by mutations R164H and I173V, known to confer ceftazidime and cefotaxime resistance individually. Therefore, these mutations might act synergistically with other cephalosporinase-conferring mutations. Residue L40 is mutated to V in clinical isolate TEM-164, or to W with another mutation known to confer ceftazidime resistance (Fujii et al., 2004), yet to our knowledge the L40F mutation has not been observed in the context of resistance. Furthermore, no resistance-conferring mutations of residue G116 have been reported. Deep-mutational scanning data (Stiffler et al., 2015) agree that its mutation does not confer a significantly adaptive phenotype suggesting that it is not a significant contributor to CTX resistance here. Despite fixation of variant cTEM-2m (**L40F**/**G116S**/T160T/**P167L**/**R178C**) after two rounds of evolution, it conferred no observable increase in resistance *in vivo* (MIC = 0.016 μg/mL, equal to cTEM-2m). This is consistent with the slight increases in MIC that result from introduction of the synergistic mutations E104K/G238S into cTEM-2m. Overall, cTEM-2m did not evolve cefotaxime resistance in *E. coli* as readily as TEM-1 and cTEM-17m.

The predominant cTEM-17m (P62P/A135A/**K158N**/R164R/**K192N**/K234K) variant conferred a 4-fold increase in MIC compared to cTEM-17m (MIC = 0.11 μg/mL). It includes two non-synonymous mutations, K158N and K192N, that have both been previously identified. K158N defines the clinical isolate TEM-127 (H158N in wild-type TEM-1) (NCBI, 2018), whereas K192N has been identified along with other mutations conferring cefotaximase activity (Salverda et al., 2010).

Overall, the most prevalent mutations in TEM-1 laboratory experiments and clinical isolates such as E104K, R164S, A237T, G238S, or E240K were not fixated. However, evolutionary path sampling did reveal previously reported mutations in cefotaximase variants. And even though it was not fixated, variants containing E104K and G238S were obtained in cTEM-17m evolution, the variant with higher slow dynamics.

## Discussion

Evolution of a new protein function often requires the initial protein to possess at least some promiscuous activity (Peisajovich and Tawfik, 2007). It has been proposed that proteins exist as an ensemble of conformers in an equilibrium that can shift through evolution to favor conformers having promiscuous function (Tokuriki and Tawfik, 2009; Maria-Solano et al., 2018). It is not known whether higher protein dynamics may facilitate modulating the conformational ensemble, potentially making a protein more amenable to evolve toward new function (Trudeau and Tawfik, 2019). Prior studies have reported changes in protein dynamics along evolutionary trajectories that were correlated with higher activity, in conjunction with the observation of epistasis in the mutations (Campbell et al., 2016; Gonzalez et al., 2016; Johansson and Lindorff-Larsen, 2018; Otten et al., 2018). Protein dynamics may thus promote non-additive interaction of mutations (Miton and Tokuriki, 2016). Increasing our understanding of the biophysical features that facilitate protein evolution finds practical applications in better directing protein engineering (Johansson and Lindorff-Larsen, 2018; Maria-Solano et al., 2018; Trudeau and Tawfik, 2019; Gardner et al., 2020; Yang et al., 2020).

We considered that TEM-1 ß-lactamase was a good model to examine the impact of initial dynamics on evolutionary trajectory because ancestral enzyme reconstruction of TEM-1 indicated that higher active-site flexibility was correlated with higher promiscuity and evolvability (Zou et al., 2015). In contrast, a study of TEM-1 variant R164S/G238S, displaying negative epistasis toward function, attributed high Ω-loop flexibility to evolutionary dead-ends (Dellus-Gur et al., 2015). Both reports point to a link between protein dynamics and evolutionary outcomes, and suggest that the relationship is complex and depend on their location and the type of protein motions.

**Figure 5:**
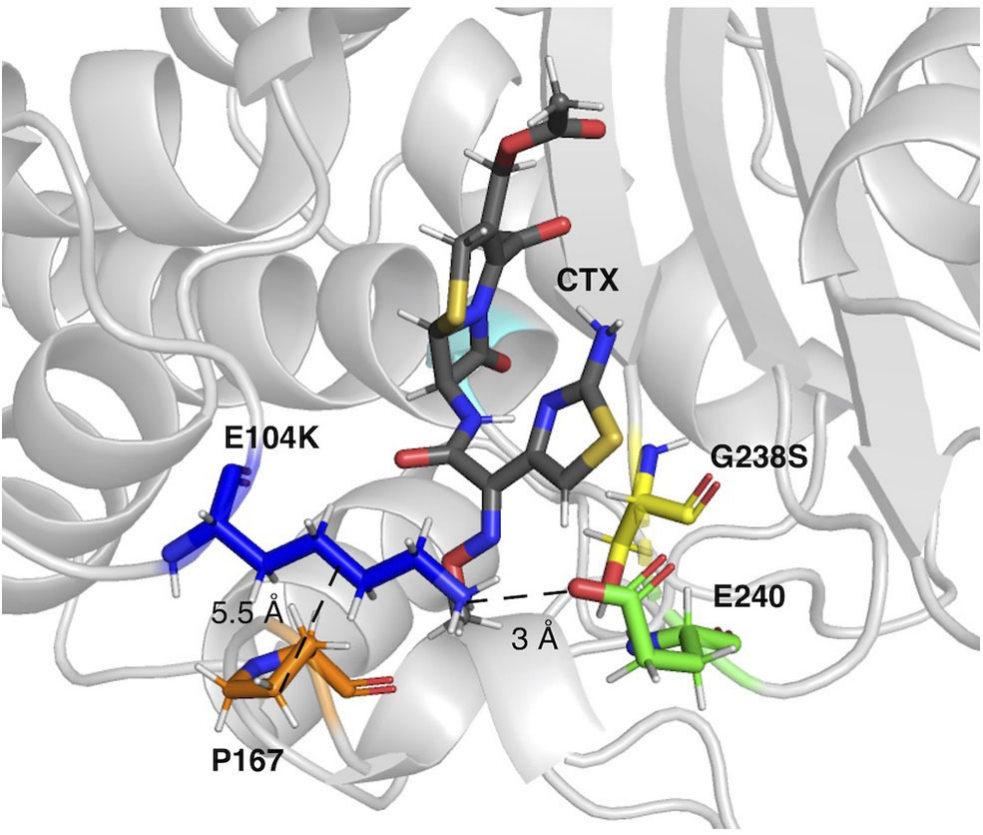
The E104K-E240 salt bridge and E104K-P167 van der Waals interaction in a representative pose of TEM-1 E104K/G238S. E104K, G238S, E240, and P167 are shown as sticks in dark blue, yellow, green, and orange, respectively. S70 is in cyan and CTX in gray.

In this study, introduction of the epistatic mutations E104K/G238S in the host ß-lactamase variants cTEM-2m and cTEM-17m maintained their synergistic effect on acquisition of *in vitro* cefotaximase activity, despite the variants' differences in protein motions at the timescale of catalytic turnover. The mechanism by which these mutations act synergistically is poorly understood (Orencia et al., 2001; Salverda et al., 2010). In fact, even the mechanism by which G238S provides such an increase in catalytic activity remains elusive (Salverda et al., 2010). It has been proposed that this mutation increases catalytic activity through a new hydrogen-bond between G238S and the oxime oxygen of cephalosporins (Raquet et al., 1995) or by enlargement of the active-site either through repositioning of the B3 ß-strand (Knox, 1995) or by repositioning of the Ω-loop (Saves et al., 1995; Cantu and Palzkill, 1998). More recently, it has been hypothesized that E104K/G238S act synergistically through long-range interactions (Miton and Tokuriki, 2016). Moreover, these mutations have been shown to reduce Ω-loop motions, stabilizing a conformer already present in TEM-1 that correlates with higher cefotaximase activity (Hart et al., 2016). Interestingly, in this first use of PELE to examine cefotaxime binding, we similarly observe the stabilization of catalytic enzyme-substrate poses in the E104K/G238S mutants. Although we did not conclusively determine the mechanism by which these mutations act synergistically, a salt bridge observed between the new E240 and K104 in all three E104K/G238S mutants has the potential to stabilize productive enzyme-substrate conformations by forming a fluctuating gate that helps to enclose the substrate within the active site cavity (Figure 5). Crystal structure analysis of ligand-free TEM-52 containing E104K/G238S and the stabilizing mutation M182T was also consistent with formation of that salt bridge: E240 was repositioned with its side-chain pointing toward E104K and the distance between those residues was decreased by 2 Å, to 4.8 Å (Orencia et al., 2001). This suggests that the gate is not triggered by substrate binding but may nonetheless contribute to keeping the substrate inside the active site. We note that the occurrence of the salt bridges trends with the improvement in catalytic efficiency of the E104K/G238S variants; cTEM-2m E104K/G238S (600-fold improvement in *k*_cat_/K_M_) displays the highest occurrence of the salt bridge (80%, Table 2). Additionally, we observed reduced distances between residue 104 and P167 in the Ω-loop of the double mutants. This has been proposed to increase the distribution of conformers able to hydrolyze cefotaxime by restricting Ω-loop motions through van der Waals contacts, thereby stabilizing the Ω-loop (Hart et al., 2016) (Figure 5). Interestingly, the Ω-loop stabilization was observed in all variants despite important slow dynamics and sequence variation occurring in the Ω-loop of cTEM-17m only (Figure 1A). The combined effects appear to contribute to the ~2-order of magnitude improvement in *k*_*cat*_ conferred by the synergistic mutations.

Considering that the double mutants of cTEM-2m and cTEM-17m show similar, strong synergy as the double mutant of WT TEM-1; that the crystal structures and NMR analysis of those two start-points for evolution are virtually indistinguishable from the WT TEM-1 (Gobeil et al., 2014, 2019); and that PELE simulations point to populations of statistically stabilized, cefotaximase-competent conformers in the double mutants, consistent with the analysis of Hart (Hart et al., 2016), we can reasonably infer that the mechanism of synergy is unchanged albeit incompletely understood in the ß-lactamase variants cTEM-2m and cTEM-17m.

The synergistic impact of mutations E104K/G238S on catalytic efficiency, on CTX resistance in *E. coli* (MIC) and on binding energy predicted upon docking CTX was most similar upon introduction into TEM-1 and cTEM-17m. This was unexpected since the latter includes 17 mutations in the active-site region relative to TEM-1 and displays vastly increased slow dynamics (Figure 1A). Despite cTEM-2m E104K/G238S displaying the strongest positive epistasis *in vitro*, with a 180-fold increase in *k*_cat_ and 600-fold improvement of *k*_cat_/K_M_, the resulting cefotaximase activity did not suffice to promote high CTX resistance in *E. coli* and yielded a lower binding energy profile. The host ß-lactamase cTEM-2m has only two mutations relative to TEM-1 and displays an intermediate extent of slow dynamics; however, those two mutations—M68L and M69T—are the immediate neighbors of the catalytic S70 and they result in a 50-fold reduction in *k*_cat_ in cTEM-2m relative to TEM-1. This low turnover rate appears to have formed an insurmountable barrier to evolutionary outcome since the inclusion of E104K/G238S in cTEM-2m, despite resulting in the greatest increase in catalytic efficiency (*k*_cat_/K_M_), did not suffice to procure CTX resistance in *E. coli*.

Similar parallels in evolutionary outcome were observed when sampling evolutionary paths in directed evolution. Mutations E104K, G238S and further mutations reported in clinical isolates of TEM-1 were selected in cTEM-17m libraries (Barlow and Hall, 2002; Salverda et al., 2010; NCBI, 2018; Palzkill, 2018). Whereas, variants selected upon directed evolution of cTEM-2m did not include prevalent cefotaximase mutations, other mutations previously identified in cefotaximase variants were reported (Fujii et al., 2004; Schenk et al., 2013). However, fixation of a cTEM-2m variant in the third round of directed evolution did not significantly increase CTX resistance as evaluated with MIC, consistent with the failure of the E104K/G238S mutations in the same context. We therefore observe similar evolutionary outcome in *E. coli* for TEM-1 and for host cTEM-17m that displays extensive slow dynamics, despite its possessing greater sequence divergence than cTEM-2m. Furthermore, the strongest positive epistasis observed *in vitro* for cTEM-2m E104K/G238S demonstrates that its differences in slow dynamics do not prevent evolvability; instead, the poor evolutionary outcome of cTEM-2m in *E. coli* appears to result from the deleterious effect of its initial mutations M68L and M69T on kinetics of cefotaxime hydrolysis.

Overall, three vastly differing patterns of protein dynamics at the timescale of catalytic turnover did not impede the high evolvability of TEM-1 ß-lactamase. Its tolerance to extensive protein dynamics at slow timescales is consistent with the robustness of TEM-1 ß-lactamases and has been hypothesized to facilitate evolution toward the recognition of new substrates (Gobeil et al., 2014, 2019; Zou et al., 2015). Whereas, loop movements and active-site cavity fluctuations occur at slow timescales and are relevant for catalysis, fast timescales where the formation and breakdown of transition states occur are also important (Henzler-Wildman and Kern, 2007). Dynamics at fast timescales are largely conserved in the three host- ß-lactamases used in this study (Figure 1A) (Gobeil et al., 2019). This backdrop of conserved fast motions and diverse slow motions provides scaffolds that have the potential to evolve toward new protein function. To our knowledge this is the first study using ß-lactamases whose dynamics landscapes have been fully characterized to explore the impact of protein motions at a specific timescale and specific regions on the evolution of new protein function and epistasis.

## Data Availability Statement

The original contributions presented in the study are included in the article/Supplementary Materials, further inquiries can be directed to the corresponding author/s.

## Author Contributions

LA and JP designed experiments and analyzed data. LA and CL-S-D performed protein expression, purification, and kinetic experiments. LA performed directed molecular evolution. FS-J designed preliminary computational simulations. VG performed computational simulations. CP provided Python scripts. LA, JP, and VG wrote the manuscript. All authors revised and approved the final version of the manuscript.

## Conflict of Interest

The authors declare that the research was conducted in the absence of any commercial or financial relationships that could be construed as a potential conflict of interest.
