# Supplementary material for: Known Evolutionary Paths Are Accessible to Engineered ß-Lactamases Having Altered Protein Motions at the Timescale of Catalytic Turnover
Source: Front Mol Biosci. 2020 Nov 20;7:599298. doi: 10.3389/fmolb.2020.599298 (PMC7716773; doi:10.3389/fmolb.2020.599298)
Supplement: Supplementary file 1 [file Table_1.DOCX]

Supplementary Material

**Sup. Table 1. Salt-bridges identified by salt-bridge plugin in VMD**

| TEM-1 | TEM-1 E104K/G238S | cTEM2m | cTEM2m E104K/G238S | cTEM17m | cTEM17m E104K/G238S |
| --- | --- | --- | --- | --- | --- |
| D163-R161 | D163-R161 | D115-HIS112 | D115-HIS112 | D115-R94 | D115-R94 |
| D176-R178 | D176-R178 | D163-R161 | D163-R161 | D115-HIS112 | D115-HIS112 |
| D179-R164 | D179-R164 | D176-R178 | D176-R178 | D157-R153 | D157-R153 |
| D214-K234 | D214-K234 | D179-R164 | D179-R164 | D163-R161 | D163-R161 |
| D233-R222 | D233-R222 | D214-K234 | D214-K234 | D176-R178 | D176-R178 |
| D254-K256 | D273-R277 | D233-R222 | D233-R222 | D179-R164 | D179-R164 |
| D273-R277 | D34-K31 | D273-R277 | D273-R277 | D214-R222 | D214-R222 |
| D34-K31 | D37-K33 | D35-K32 | D35-K32 | D233-R222 | D233-R222 |
| D37-K33 | D49-K54 | E121-R94 | E166-K73 | D233-K215 | D233-K215 |
| D49-K54 | E121-R94 | E166-K73 | E171-R164 | D254-K256 | D254-K256 |
| E121-R94 | E166-K73 | E171-R164 | E171-R241 | D273-R277 | D273-R277 |
| E166-K73 | E171-R164 | E171-R241 | E177-R65 | D35-K32 | D35-K32 |
| E171-R164 | E171-R178 | E177-R65 | E197-R204 | E110-K111 | E110-K111 |
| E171-R178 | E177-R65 | E197-R204 | E240-R241 | E166-K73 | E121-HIS112 |
| E177-R65 | E197-R204 | E240-R241 | E240-K104 | E171-R164 | E166-K73 |
| E197-R204 | E240-K104 | E274-R277 | E274-R277 | E240-R241 | E171-R164 |
| E281-R277 | E281-R277 | E281-R277 | E281-K32 | E37-R61 | E240-R241 |
| E281-K31 | E281-K31 | E281-K32 | E37-R61 | E37-K34 | E240-K104 |
| E36-R61 | E36-R61 | E37-R61 | E37-K34 | E48-R259 | E37-R61 |
| E47-R259 | E47-R259 | E37-K34 | E48-R259 | E64-R43 | E37-K34 |
| E64-R42 | E64-R42 | E48-R259 | E64-R43 | E64-R61 | E48-R259 |
| E64-R61 | E64-R61 | E64-R43 | E64-R61 | E89-R83 | E64-R43 |
| E89-R83 | E89-R83 | E64-R61 | E89-R83 | E89-R93 | E64-R61 |
| E89-R93 | E89-R93 | E89-R83 | E89-R93 |  | E89-R83 |
|  |  | E89-R93 |  |  | E89-R93 |

**Sup. Table 2. Mutations identified in clones prior to selection at each generation of evolution.**

|  | **cTEM2m** | **cTEM17m** |
| --- | --- | --- |
| **Generation 1** | A9V, K111M, N136S, K146* | WT |
|  | Y46C, P62P, T140I | WT |
|  | R43*, V108L | I127L |
|  | I47L, S124N, T128S | V89I, P175A |
|  | WT | F81I, V89E, L160I |
|  | L57L | R60H, C132G, L145I |
|  | L57L, K215I | F15L, Q97H |
|  | K234L | L57P, E62D, I50F |
|  | WT | N79S, F81L, L111* |
|  | L40S | F81I, H105Y |
|  |  | A11T |
|  |  | I163N |
|  |  | WT |
|  |  | WT |
|  |  | WT |
|  |  | WT |
|  |  |  |
| **Generation 2** | WT | L57L, P62P |
|  | D115G, N170I | A215A |
|  | F17S, | E104D, D163D |
|  | WT | P62P, L162L |
|  | F17S, G116S, T133I | L162L, T182T |
|  | Q24*, A232A, R128H | G54G, A125A, L207S |
|  | C77S, E104V, T118A, E178D, A184A | E64*, K192E |
|  | E28*, T48T, P55L, R66C | E64*, V84L |
|  | D35N, L102M, G116S | D168N, I282I |
|  | E28*. T133P | R275R |
|  | G156D, P167L, R178C, R241H |  |
|  | D232D, A270T |  |
|  | T18S |  |
| **Generation 3** | L40F, I56F | A36A, P62P, A135V |
|  | L30P, L40F, C77R | E64*, A125A, |
|  | I7F, L31L, L40F, G45R | S59C, E64*, M117L, R164R |
|  | K2R, L40F, G116S | A15T, E64*, A125A |
|  | T29T, L91V, G116S | E64*, S70S, R164R |
|  | L40F, I95L, G116S, T160T, R178C | A15T, P62P, I95V, |
|  | G54C, G116S, T160T | E64*, A135V, T141R, R164R |
|  | L40F, G116S, T160T, P167L, R178C | P62P, T118T |
|  | L40F, C77*, P107P, K111N, G116S, T160T, P167L, R178C | Q39L, E64* |
|  | K2R, L40F, G116S, T160T, P167L, R178C | T21S, N52S, E64*, A135V, R164R |
|  | R81R, S98Y, E104V, T114A, T118A, S124N. T160T, P167L, R178C |  |
|  | L12L, G14D, L40F, G116S, T160T, P167L, R178C, S223P |  |

*represents stop codons
